# Cardiac Autonomic Dysfunction Is Associated With Risk of Diabetic Kidney Disease Progression in Type 2 Diabetes Mellitus

**DOI:** 10.3389/fendo.2022.900465

**Published:** 2022-07-01

**Authors:** Haixia Zeng, Jianmo Liu, Zheng Chen, Peng Yu, Jianping Liu

**Affiliations:** Department of Endocrinology and Metabolism, The Second Affiliated Hospital of Nanchang University, Nanchang, China

**Keywords:** T2DM, HRV, DKD, ACR, eGFR

## Abstract

**Background:**

Evidence on the relationship between heart rate variability (HRV) and albumin-to-creatinine ratio (ACR) combined with estimated glomerular filtration rate (eGFR) in patients with type 2 diabetes mellitus (T2DM) is rare. Thus, this study aimed to investigate the relationship between heart rate variability and the risk of diabetic kidney disease (DKD) progression in diabetes patients.

**Method:**

Overall, 747 T2DM patients who were admitted to the Second Affiliated Hospital of Nanchang University underwent 24-hour dynamic electrocardiograms for HRV analysis. Time-domain HRV measures included mean heart rate, standard deviation of the R-R interval (SDNN), SDNN index, root mean squared difference of successive RR intervals (RMSSD), and percent of adjacent RR intervals with a difference greater than 50 ms (PNN50). Frequency-domain measures included low frequency (LF), very low frequency (VLF), high frequency (HF) components and LF-to-HF ratio. The risk of DKD progression was determined by combining ACR and eGFR and stratified as low risk (Group A), moderately increased risk (Group B), high risk (Group C), and very high risk (Group D) based on the Kidney Disease: Improving Global Outcomes guidelines.

**Result:**

There were significant differences in HRV parameters among the four risk groups (SDNN: 113 ms vs 109 ms vs 101 ms vs 81 ms, *P*<0.01; LF: 240.2 ms^2^ vs 241.1 ms^2^ vs 155.2 ms^2^ vs 141.9 ms^2^, *P*<0.01; LF-to-HF ratio: 1.70 vs 1.24 vs 1.12 vs 0.93, *P*<0.01; VLF: 723.7 ms^2^ vs 601.1 ms^2^ vs 446.4 ms^2^ vs 356.3 ms^2^, *P*<0.01). A very high risk of DKD progression was significantly associated with a lower SDNN (β=-19.5, 95% CI: -30.0 to -10.0, *P*<0.01), and moderately increased, high, and very high risks were associated with lower LF-to-HF ratio and VLF (*P*<0.05). Logistic regression analysis showed that group D had a higher risk of reduced SDNN, LF-to-HF ratio, and VLF compared with group A after adjusting for systolic blood pressure, glycated haemoglobin, haemoglobin, high-density lipoprotein cholesterol, and age (odds ratio (95% CI): 0.989 (0. 983–0.996), 0.674 (0.498–0.913), and 0.999 (0.999–1.000), respectively).

**Conclusion:**

Cardiac autonomic dysfunction is associated with a risk of DKD progression in adults with T2DM, and reduced heart rate variability increased such risk. Thus, HRV screening may be necessary in patients with T2DM, especially those with high proteinuria.

## Introduction

Cardiac autonomic neuropathy (CAN)is a common chronic complication of diabetes, occurring in 25%–75% of patients with type 2 diabetes (T2DM). CAN mainly presents as resting tachycardia, orthostatic hypotension, syncope, and cardiac dysfunction (1). However, CAN may remain in a subclinical state for several years; the patient may be asymptomatic, and a decrease in heart rate variability (HRV) may only be found on physical examination. HRV is a non-invasive indicator for detecting and quantifying CAN in diabetes patients, reflecting the dynamic balance of the sympathetic and parasympathetic nervous systems in regulating cardiovascular function. Further, the HRV test has higher sensitivity for detecting cardiac autonomic changes than conventional tests based on dynamic cardiovascular operations, thus making it helpful for the early diagnosis of CAN (2, 3).

Albuminuria is considered to be a marker of systemic endothelial dysfunction in diabetes patients, and early detection of microalbuminuria can prevent end-stage renal disease and cardiovascular complications of diabetes. Previous studies have shown that CAN is associated with higher albumin-to-creatinine ratio (ACR) and poor glycaemic control (4, 5). Further, a decrease in estimated glomerular filtration rate (eGFR) was found to be strongly associated with an increased risk of cardiovascular disease and death (6). However, evidence on whether cardiac autonomic function is associated with renal dysfunction based on changes in microalbuminuria and glomerular filtration rate is rare. The Kidney Disease: Improving Global Outcomes (KDIGO) recommends combining chronic kidney disease (CKD) staging and albuminuria category to assess the risk of progression of diabetic kidney disease (DKD) (7). Although CAN is a risk factor for progression of kidney disease in diabetes patients (8, 9), there are few studies on the relationship of HRV with ACR combined with eGFR in patients with T2DM. Therefore, this study aimed to investigate the relationship of HRV with the risk of DKD progression. Towards this goal, the risk of progression of nephropathy in T2DM patients was classified according to CKD stage and albuminuria category, and HRV was measured.

## Material and methods

### Study Design and Patients

This retrospective cohort study was approved by the Information Management Organization of the Second Affiliated Hospital of Nanchang University, and the need for informed consent was waived owing to the retrospective nature of the study. We included T2DM patients (mean age: 68.6 ± 11.5 years [range: 34–92 years]) who were hospitalised between January 2017 and October 2021. The exclusion criteria were type 1 diabetes mellitus, nephrotic syndrome, presence of active urinary sediment (erythrocytes, leukocytes, or cell casts), refractory hypertension, and incomplete data. After excluding 98 patients, 747 T2DM patients (337 males and 410 females) were included the analysis. The diagnosis and history of T2DM were carefully reviewed by at least two authors. Incident T2DM was defined as a diagnosis of diabetes after discharge and glycated haemoglobin (HbA1c) ≥ 6.5% or fasting blood glucose ≥ 7 mmol/L or random blood glucose ≥ 11.1 mmol/L, or have received insulin or oral hypoglycaemic drug treatment. Data were obtained from the Big Data Centre Management System/Electronic Medical Record Cohort Database of the Second Affiliated Hospital of Nanchang University.

### Assessments

Height and weight were measured by standard methods, and body mass index (BMI) was calculated by dividing weight (kg) by the square of height (m). Blood pressure was measured using OMRON HEM-7130 blood pressure monitor with an appropriately sized cuff. Blood and urine samples were obtained after an overnight fast within 0 to 5 days prior to 24-hour dynamic electrocardiograms. All blood and urine samples were analysed at the Laboratory Centre of the Second Affiliated Hospital of Nanchang University. Lipid profile measurements (cholesterol, high-density lipoprotein cholesterol, low-density lipoprotein cholesterol, triglycerides) were measured using Automatic biochemical analyser (Cobas c800, Roche, Switzerland).

Whole blood haemoglobin was detected with a whole blood cell analyser (Bc6800, Mindray, Xiamen Haifei Biotechnology Co., Ltd, China), and HbA1c was measured using high-performance liquid chromatography (4500MD, AB SCIEX, USA). Urine albumin was measured using laser immunonephelometry (Siemens BN ProSpec), and urine creatinine was measured using a chromatographic stable isotope dilution electrospray mass spectrometry-mass spectrometry method. ACR (mg/mmol) was calculated as the urine albumin concentration (mg/L) divided by the urine creatinine (mmol/L). eGFR was calculated using the formula: eGFR (mL/min/1.73 m^2^) = 42 ×height (cm)/plasma creatinine (µmol/L).

The patients underwent a 24-hour continuous electrocardiogram recording in the supine position, using the Plus-type three-channel DCG of the American DMS company. The measurement recording started from 8–9 am and ended at 8–9 am the next day.

### Variable Definitions

HRV was defined as the variations of heart rate and successive cardiac cycles under the control of the autonomic nervous system. Time-domain measures of overall HRV included the mean heart rate, standard deviation of the R-R interval (SDNN), SDNN index, root mean squared difference of successive RR intervals (RMSSD), and the percentage of adjacent RR intervals with a difference greater than 50 ms (PNN50). Frequency-domain measures included low-frequency (LF), very low-frequency (VLF), high-frequency (HF) components, and the LF-to-HF ratio, considered to be an estimate of the relative sympathetic and parasympathetic balance.

**Figure 1:**
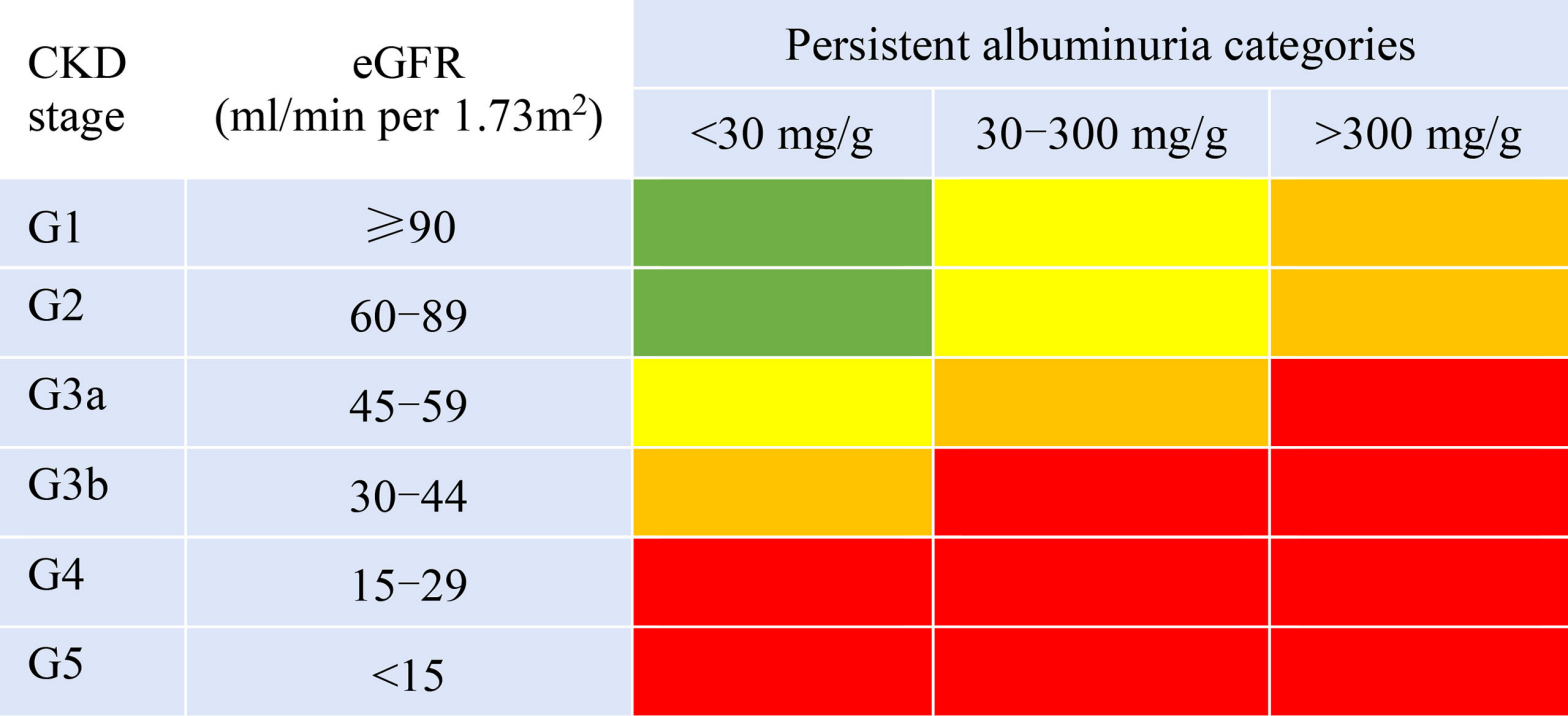
Prognosis of CKD by eGFR and albuminuria categories. Green areas represent low risk; yellow, moderately increased risk; orange, high risk; and red, very high risk.

ACR combined with eGFR was used to stratify the risk of DKD progression following the KDIGO guidelines (10). The patients were classified into four risk groups: low risk (Group A), moderately increased risk (Group B), high risk (Group C), and very high risk (Group D) (**Figure 1**).

### Statistical Analysis

Normally distributed continuous variables were compared using one-way analysis of variance, summarised as mean ± SD for parametric data. Meanwhile, non-normal distributed variables expressed as the median with interquartile range, and categorical variables were presented as the number (percentage). Correlations between two variables were assessed using Spearman correlations. Multivariate linear regression and logistic regression analyses were used to determine the association between HRV and the risk of renal disease progression. One-way analysis of variance was used to establish whether HRV differed according to the risk of DKD progression category. If significant differences were found, individual groups were compared using the Bonferroni *post hoc* test. All statistical analyses were conducted using SPSS version 25 software (SPSS Inc., Version 25.0, Chicago, IL, USA). *P*<0.05 was considered statistically significant.

## Results

The mean patient age was 68.6 ± 11.5 years. Groups A, B, C, and D included 409, 163,77, and 98 patients, respectively. There were no significant differences in sex, height, weight, BMI, and average heart rate among the four groups. Except for high-density lipoprotein cholesterol, the other lipid profiles showed no significant difference. The risk of DKD progression increased with age. Participants with higher risk of DKD progression also had significantly higher systolic blood pressure (SBP) (*P*<0.01) and significantly lower whole blood haemoglobin (*P*<0.01) (**Table 1**).

**Table 1:** Patient characteristics by DKD progression risk.

	Group A	Group B	Group C	Group D	*P*
Sex (female, male)	216 (53%), 193 (47%)	98 (60%), 65 (40%)	45 (58%), 32 (42%)	51 (52%), 47 (48%)	0.41
Age (years)	66.0 (10.9)	70.0 (11.7)	72.6 (11.7)	74.0 (10.2)	<0.01
Height (cm)	156 (162–170)	158 (164.5–170)	156 (163–170)	155 (162–168)	0.24
Weight (kg)	58 (65–73)	60 (66–75)	59.5 (64–73)	59 (64.5–70)	0.37
BMI (kg/m²)	22.6 (24.6–26.8)	22.9 (24.8–26.8)	23.0 (24.6–27.2)	22.4 (24.5–25.8)	0.53
SBP (mmHg)	135.2 (20.3)	142.2 (22.7)	145.3 (22.7)	146.2 (26.0)	<0.01
DBP (mmHg)	77.2 (12.4)	77.1 (14.3)	79.2 (16.0)	77.9 (15.5)	0.68
HbA1c (%)	7.27 (1.65)	7.96 (1.91)	8.05 (1.80)	7.36 (1.67)	<0.01
HDL-C (mmol/L)	1.07 (0.93–1.26)	0.98 (0.84–1.18)	0.97 (0.83–1.22)	1.03 (0.84–1.19)	<0.01
NHDL-C(mmol/L)	3.2 (2.5–4.1)	3.2 (2.4–4.1)	3.0 (2.4–4.2)	3.3 (2.3–4.3)	0.9
LDL-C (mmol/L)	2.5 (1.9–3.2)	2.5 (1.8–3.2)	2.3 (1.8–3.0)	2.5 (1.8–3.2)	0.77
Triglycerides(mmol/L)	1.4 (1.0–2.0)	1.4 (1.0–2.1)	1.4 (1.0–2.2)	1.4 (1.0–2.0)	0.69
Cholesterol (mmol/L)	4.4 (3.5–5.3)	4.2 (3.4–5.2)	4.0 (3.4–5.1)	4.5 (3.5–5.5)	0.69
Hb (g/L)	134 (123–144)	132 (121–145)	127 (114–137)	112 (97–123)	<0.01

Data are presented as the mean (SD), median (IQR), or n (%).

SBP, systolic blood pressure; DBP, diastolic blood pressure; HDL-C, HDL cholesterol; NHDL-C, NHDL cholesterol; LDL-C, LDL-cholesterol; Hb, haemoglobin

ACR as a continuous variable was negatively correlated with SDNN (r=-0.21, *P <*0.01), SDNN Index (r=-0.16, *P*<0.01), HF (r=-0.08, *P* = 0.03), LF (r=-0.15, *P*<0.01), LF/HF (r=-0.15, *P*<0.01), and VLF (r=-0.28, *P*<0.01). Meanwhile, eGFR was positively correlated with SDNN (r=0.16, *P*<0.001), LF (r=0.12, *P*<0.01), LF/HF (r=0.25, *P*<0.01), and VLF (r=0.27, *P*<0.01) (**Table 2**).

**Table 2:** Correlation between renal function parameters and HRV parameters.

HRV parameter	ACR (mg/g)		eGFR (ml/min per 1.73m^2^)	
	*r*	*P*	*r*	*P*
HR (bpm)	0.10	<0.01	0.01	0.87
SDNN (ms)	−0.21	<0.01	0.16	<0.01
SDNN Index	−0.16	<0.01	0.07	0.07
RMSSD (ms)	−0.05	0.18	−0.06	0.09
pNN50 (%)	−0.07	0.06	−0.04	0.23
HF (ms^2^)	−0.08	0.03	−0.01	0.97
LF (ms^2^)	−0.15	<0.01	0.12	<0.01
VLF (ms^2^)	−0.28	<0.01	0.27	<0.01
LF/HF	−0.15	<0.01	0.25	<0.01

Univariable analysis with Spearman correlation is shown.

HRV, heart rate variability. eGFR, estimated glomerular filtration rate. ACR, albumin-to-creatinine ratio. LF/HF, LF-to-HF ratio.

**Figure 2:**
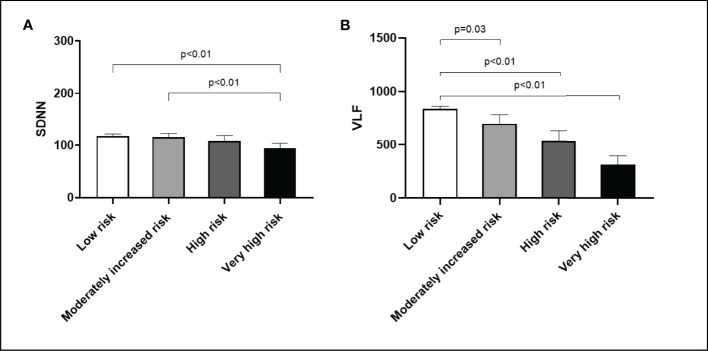
DKD progression risk with respect to heart rate variability. **(A)** SDNN. **(B)** VLF. Bar (one bar per column) plots are shown in **(A, B)** The *P* values are calculated using Bonferroni *post hoc* test after significant one-way ANOVA.

The risk of DKD progression was significantly associated with SDNN, VLF, and LF/HF in the multivariate linear regression analysis. The reduction of VLF was closely related to a moderately increased risk (β=-99.4, 95% CI: -197.5 to -1.3, *P*<0.04), high risk (β=-237.1, 95% CI: -369.2 to -104.9, *P*<0.01), and very high risk (β=-193.7, 95% CI: -320.8 to -66.6, *P*<0.01) of DKD progression. It was also independently related to HbA1c and haemoglobin levels. In addition, decreased LF/HF was also closely related to a moderately increased risk (β=-0.29, 95% CI: -0.49 to -0.09, *P*<0.01), high risk (β=-0.29, 95% CI: -0.56 to - 0.02, *P*=0.03), and very high risk (β=-0.38, 95% CI: -0.64 to -0.12, *P*<0.01) of DKD progression. It was also independently associated with SBP and haemoglobin levels. Similar results were found for SDNN, although only the difference between group A and D reached significance (**Table 3** and **Figure 2**).

**Table 3:** Multivariable analysis of heart rate variability outcomes and risk of DKD progression.

HRV parameter	Factor	β (95% CI)	*P*
	Group B	0.96 (−7.1 to 9.0)	0.82
	Group C	−6.1 (−17.0 to 4.8)	0.27
	Group D	−19.5 (−30.0 to −10.0)	<0.01
	HbA1c	−3.3 (−5.1 to −1.5)	<0.01
SDNN	SBP	−0.2 (−0.4 to −0.1)	<0.01
	Group B	−99.4 (−197.5 to −1.3)	0.04
	Group C	−237.1 (−369.2 to −104.9)	<0.01
	Group D	−193.7 (−320.8 to −66.6)	<0.01
	HbA1c	−23.2 (−45.2 to −1.1)	0.04
VLF	Hb	3.6 (1.4 to 5.9)	<0.01
	Group B	−0.29 (−0.49 to −0.09)	<0.01
	Group C	−0.29 (−0.56 to −0.02)	0.03
	Group D	−0.38(−0.64 to −0.12)	<0.01
	SBP	0.01 (0.00 to 0.01)	<0.01
LF/HF	Hb	0.02 (0.01 to 0.02)	<0.01

BMI, age, and lipid profile are not significant explanatory variables in the above.

models. +Group A was set as the reference group. HRV, heart rate variability.

The HRV parameters SDNN, SDNN index, LF, LF/HF, and VLF decreased with increasing risk of DKD progression (*P*<0.01) (**Table 4**). Further multivariate logistic regression analysis adjusted for SBP, HbA1c, haemoglobin, HDL-cholesterol, and age showed that compared with group A, group D had a higher risk of reduced SDNN, LF/HF, and VLF (odds ratio (95% CI): 0.989 (0.983–0.996), 0.674 (0.498–0.913), and 0.999 (0.999–1.000), respectively) (**Table 5**).

**Table 4:** Heart rate variability stratified by DKD progression risk.

HRV parameter	Group A	Group B	Group C	Group D	*P*
HR (bpm)	72 (65–79)	73 (64–82)	73 (65–84)	73.5 (66–81)	0.38
SDNN (ms)	113 (88–144)	109 (84–141)	101 (76–124)	81 (64–122)	<0.01
SDNN Index	45 (35–58)	45 (32–51)	40 (27–73)	38 (27–53)	<0.01
RMSSD (ms)	27 (21–37)	30 (22–46)	28 (17–50)	26 (20–47)	0.34
pNN50 (%)	5 (2–13)	7 (2–20)	5 (2–21)	4(1–20)	0.18
HF (ms^2^)	130.1 (70.3–263.5)	145.2 (67.1–439.8)	143.3 (47.2–517.0)	123.3 (45.5–388.2)	0.53
LF (ms^2^)	240.2 (134.3–430.0)	241.1 (103.7–442.1)	155.2 (79.2–536.6)	141.9 (49.5–387.5)	<0.01
LF/HF	1.70 (1.08–2.41)	1.24 (0.69–3.08)	1.12 (0.54–3.24)	0.93 (0.54–1.69)	<0.01
VLF (ms^2^)	723.7 (483.9–1112.4)	601.1 (377.4–863.9)	446.4 (184.5–745.9)	356.3 (203.8–630.4)	<0.01

HRV, heart rate variability. HR, heart rate.

**Table 5:** Multivariate analysis of risk factors for moderately increased, high, and very high risks of DKD progression.

HRV parameter		Group B		Group C		Group D	
		OR (95% CI)	*P*	OR (95% CI)	*P*	OR (95% CI)	*P*
SDNN	Model 1	1.001 (0.997–1.005)	0.697	0.997 (0.991–1.003)	0.386	0.990 (0.983–0.996)	0.003
	Model 2	1.000 (0.996–1.005)	0.822	0.997 (0.991–1.003)	0.299	0.989 (0.983–0.996)	0.002
SDNN Index	Model 1	1.003 (0.998–1.008)	0.220	1.004 (0.998–1.011)	0.186	1.002 (0.995–1.008)	0.604
	Model 2	1.002 (0.997–1.007)	0.500	1.002 (0.996–1.009)	0.523	1.000 (0.994–1.007)	0.980
LF	Model 1	1.000 (1.000–1.001)	0.087	1.000 (1.000–1.001)	0.053	1.000 (1.000–1.001)	0.261
	Model 2	1.000 (1.000–1.001)	0.177	1.000 (1.000–1.001)	0.157	1.000 (1.000–1.001)	0.463
LF/HF	Model 1	0.770 (0.640–0.926)	0.005	0.758 (0.586–0.980)	0.034	0.607 (0.452–0.815)	0.001
	Model 2	0.834 (0.690–1.009)	0.061	0.879 (0.675–1.146)	0.342	0.674 (0.498–0.913)	0.011
VLF	Model 1	1.000 (0.999–1.000)	0.037	0.999 (0.998–0.009)	<0.001	0.999 (0.999–1.000)	0.006
	Model 2	1.000 (0.999–1.000)	0.073	0.999 (0.998–1.000)	0.002	0.999 (0.999–1.000)	0.017

Model 1: adjusted for SBP, Hb, and HbA1c.

Model 2: model 1 adjustments+age and HLD-C.

+Group A was set as the reference group.

CI, confidence interval; OR, odds ratio.

## Discussion

The causal relationship between CAN and CKD is still unclear, and varying study populations and methods of CAN assessment may lead to different results. This study found that cardiac autonomic dysfunction is associated with the risk of nephropathy progression in patients with T2DM. Compared with those at low risk of DKD progression, patients with a very high risk of DKD progression had lower overall HRV (SDNN, VLF, LF/HF), and this association was independent of age, HbA1c, SBP, high-density lipoprotein cholesterol, and whole blood haemoglobin.

A study of non-diabetes patients found that although HRV reduction was not an independent risk factor for CKD, it was independently associated with eGFR reduction at baseline in patients with CKD. This suggests that CAN may be a complication rather than a cause of CKD (11). However, albuminuria, CKD, and CAN are all common microvascular complications with similar pathogenic mechanisms driven by hyperglycaemia and other risk factors in patients with diabetes. The relationship between CAN and CKD has been reported in some studies of patients with T2DM (12, 13). SDNN reflects the total tension of sympathetic and parasympathetic nerves and can be used to evaluate the overall regulation of cardiac autonomic nervous system (14). Meanwhile, HF is associated with parasympathetic activity (15), and LF is associated with both sympathetic and parasympathetic activity (16). VLF is an indicator of sympathetic activity, and the LF-to-HF ratio is considered to be an estimate of the relative sympathetic and parasympathetic balance (17).

Glomeruli and renal tubules are mainly innervated by sympathetic nerves, and changes in sympathetic nerve activity and imbalance of autonomic nerve function can affect renal homeostasis (renal vascular resistance, filtration) and accelerate the decline of renal function (13, 18). On the one hand, CAN can change the circadian rhythm of blood pressure, reduce the drop of blood pressure at night, and increase the blood flow and load of the kidney, which is an important cause of kidney damage (19). On the other hand, sympathetic nerve endings directly innervate the kidney, enhance the absorption of solutes and fluids, and affect the renal tubular function (20). Either increased sympathetic nerve activity or simultaneous changes in sympathetic and parasympathetic activity can be associated with reduced HRV and poor renal prognosis (17). The results of our study showed that the reduction of SDNN, LF/HF, and VLF was closely related to the increased risk of DKD progression. Compared with the low risk group, the very high-risk group of DKD progression had significantly lower SDNN, LF/HF, and VLF. Our findings are consistent with those of two other studies of type 1 diabetes patients that found a significantly faster progression of renal dysfunction in the HRV-poor group (4, 21).

There may be potential autonomic crosstalk between the kidneys and the cardiovascular system. Autonomic dysfunction as a broad term mainly refers to a condition in which tonic and reflex control of autonomic outflows is altered, favouring increased sympathetic nerve activity and depressed parasympathetic function (22). Autonomic dysfunction is associated with increased oxidative stress and elevated levels of proinflammatory cytokines (23, 24). Several studies suggest that oxidative stress plays a role in the development of CAN in diabetic patients (25, 26). Kidney disease is associated with a decrease in antioxidant scavenging enzymes and increase in the production of reactive oxygen species (ROS), thereby leading to elevated oxidative stress (27). Furthermore, the accumulation of reactive ROS is important determinants that promote the DKD progresses (28). Studies in DKD rats show that inhibition of ROS and nucleotide binding and oligomerization domain-like receptor family pyrin domain-containing 3 (NLRP3) inflammasome possesses kidney protection effects including attenuating urinary microalbumin excretion, and mitigating renal histopathological lesions on DN (29). Cardiac inflammation is an important pathological process involved in sympathetic overactivation induced cardiac injury (30). Sympathetic overactivation induces cardiac inflammation and dysfunction by activating the NLRP3 inflammasome in cardiomyocytes (31). However, it remains to be determined whether Oxidative stress and proinflammatory cytokines involved in sympathetic overactivation promotes DKD progression and altered cardiovascular autonomic function.

Obesity is associated with decreased parasympathetic nerve activity and increased sympathetic nerve activity (32, 33). The present study found no differences in BMI between the four risk groups for DKD progression, helping to rule out confounding effects of BMI on HRV and risk of DKD progression. However, BMI is weakly correlated with cardiac autonomic function indicators (33), and thus, more sensitive indicators of subcutaneous and visceral obesity should be incorporated in the future. Additionally, SBP increased with increased risk of kidney disease progression in the current study, whereas there was no significant difference in DBP. In the VADT study, baseline SBP was associated with decreasing eGFR and worsening ACR among veterans with T2DM, whereas DBP levels were not significantly associated with risk of renal outcomes (34). Similar conclusions were also obtained in the Australian Obesity Diabetes Study, wherein there was no direct relationship between DBP and renal outcome. However, higher pulse pressure was a significant risk factor for decreased eGFR (35). Elevated blood pressure also induces decreased parasympathetic activity and increased sympathetic activity, and elevated circadian systolic blood pressure gradients are significantly associated with autonomic dysfunction in hypertension patients (36, 37). Similarly, we found that SDNN and LF/HF reduction were closely related to SBP.

A limitation of this study was its cross-sectional design, and thus, causal and temporal relationships between cardiac autonomic function and risk of DKD progression could not be examined. Autonomic dysfunction and CKD progression may share common aetiologies such as obesity, inflammation, insulin resistance, and genetic factors (38–42), but these were not fully measured in this study.

In conclusion, reduced HRV in patients with T2DM is associated with DKD and its risk of progression. Thus, screening for HRV may be necessary in patients with T2DM, especially those with high proteinuria.

## Data Availability Statement

The original contributions presented in the study are included in the article/supplementary material. Further inquiries can be directed to the corresponding author.

## Ethics Statement

Written informed consent was not obtained from the individual(s) for the publication of any potentially identifiable images or data included in this article.

## Author Contributions

HZ contributed to execution of the study, completed the data analysis, and wrote the article. JL contributed to critically revised the article and approved the final version. All authors contributed to the article and approved the submitted version.

## Conflict of Interest

The authors declare that the research was conducted in the absence of any commercial or financial relationships that could be construed as a potential conflict of interest.

## Publisher’s Note

All claims expressed in this article are solely those of the authors and do not necessarily represent those of their affiliated organizations, or those of the publisher, the editors and the reviewers. Any product that may be evaluated in this article, or claim that may be made by its manufacturer, is not guaranteed or endorsed by the publisher.
